# Superior Mesenteric Artery Syndrome: A Forgotten Cause of Duodenal Obstruction

**DOI:** 10.7759/cureus.10710

**Published:** 2020-09-29

**Authors:** Asim Haider, Madhav Sharma, Ayesha Siddiqa

**Affiliations:** 1 Internal Medicine, BronxCare Health System, Bronx, USA

**Keywords:** superior mesenteric artery syndrome, small bowel obstruction, weight loss, metastatic breast cancer

## Abstract

Superior mesenteric artery (SMA) syndrome has been described in medical literature as a rare cause of duodenal occlusion. It has a varied presentation, with distressing gastrointestinal symptoms such as nausea, abdominal pain, and further weight loss. Several conditions contribute to duodenal obstruction in SMA syndrome. We present a case of SMA syndrome in a patient with malignant breast cancer who presented with sudden onset of severe nausea and voluminous vomiting. Various imaging studies revealed a distended proximal intestine with a transition point in the third part of the duodenum. The patient was managed conservatively with nasogastric decompression and fluid electrolyte management, leading to symptomatic relief.

## Introduction

Rokitansky, in 1842, was the first one to describe the superior mesenteric artery (SMA) syndrome [1]. Since then, it has been reported extensively in the medical literature either as an incidental radiologic finding or secondary to a condition causing significant weight loss presenting with distressing gastrointestinal symptoms. SMA syndrome develops due to compression of the third part of the duodenum between the aorta and the SMA, mainly due to the loss of fat pad buffer between the two arteries. The most common presenting complaints are nausea, intractable emesis, abdominal pain, and weight loss. The penultimate event triggering the occlusion of the duodenum is one of the medical conditions associated with rapid weight loss, such as anorexia nervosa, inflammatory conditions, such as tuberculosis and brucellosis, malignancy, and rapid weight loss after bariatric surgery or a drug administration. Imaging studies are essential to make a diagnosis. The management can be conservative or surgical depending upon the acuity of clinical presentation, etiology, and response to conservative management.

## Case presentation

**Figure 1:**
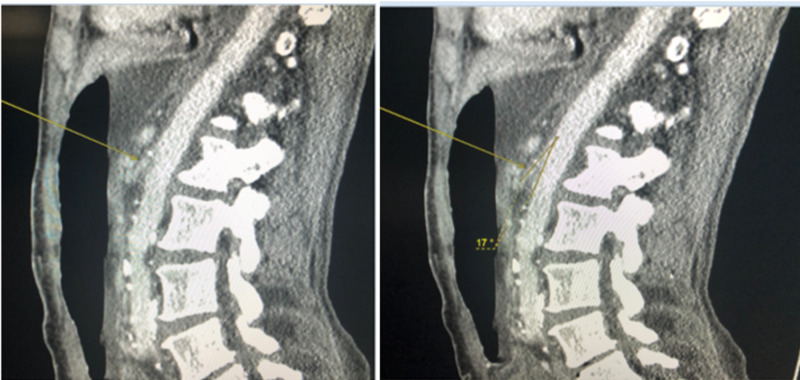
Contrast-enhanced computed tomography of the abdomen showing the origin of the superior mesenteric artery (left side) and an aortomesenteric artery angle of 17 degrees (right side)

A 65-year-old African American female with a medical history of metastatic left breast invasive ductal carcinoma (poorly differentiated with metastasis to lungs and chest wall), malignant left pleural effusion, hypertension, hyperlipidemia, aortic stenosis, cerebrovascular accident with residual left-sided weakness, and sinus bradycardia presented to the emergency department with symptoms of shortness of breath associated with multiple episodes of bilious, non-bloody vomiting for one day. She reported that she had been throwing up the whole day, and her appetite had been poor for over one month. She denied any abdominal pain. Her weight upon presentation was 103 pounds and was 115 pounds two months ago. Her initial vital signs were as follows: pulse 96 beats per minute, the respiratory rate per minute, blood pressure 121/85 mm Hg, and temperature 98 F. She was found to be hypoxic, saturating 70% on room air. On abdominal examination, the abdomen was soft, non-tender, non-distended, and without any palpable masses. There was no guarding, rebound tenderness, or organomegaly. The patient was put on bi-level positive airway pressure (BiPAP) for the stabilization of respiratory symptoms. Routine blood and urine examination were normal. Chest X-ray showed worsening left-sided pleural effusion, and bilateral interstitial infiltrates. A computed tomography (CT) scan of the abdomen and pelvis with contrast material showed a distended stomach and duodenum. Also, a transition point was noted near the aorta, which suggested the occlusion of the duodenum by the SMA. The aortomesenteric artery angle was 17 degrees (Figure 1).

**Figure 2:**
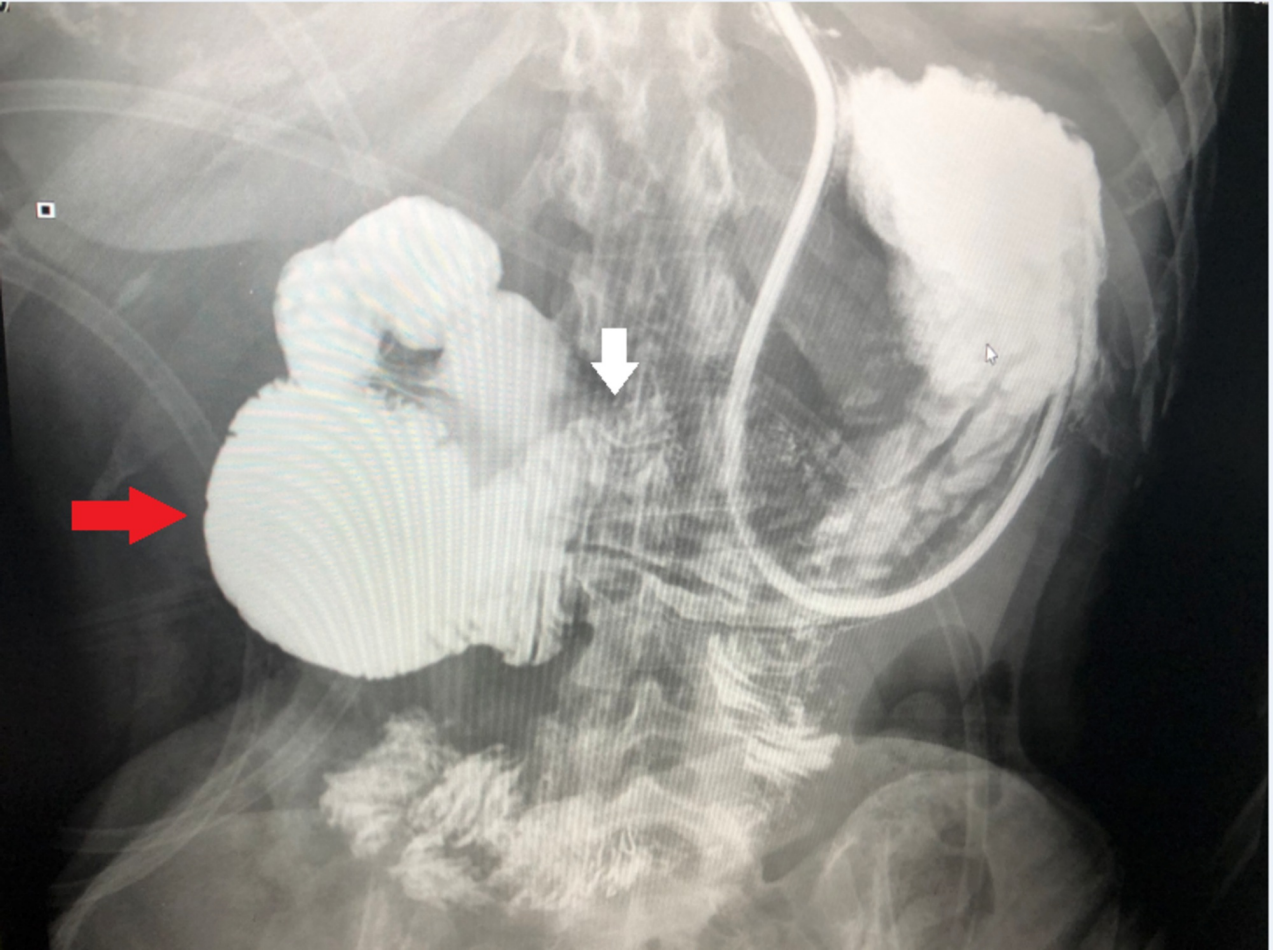
X-ray upper gastrointestinal series with water-soluble contrast showing the transition point; distended duodenum (red arrow) followed by partially obstructed mid-third duodenum (white arrow)

The CT scan was followed by the upper gastrointestinal series, which showed that the duodenal loop was dilated to the mid-point of the third portion, beyond which the slowest flow of barium was seen into the remainder of the jejunum and ileum. Normal diameter jejunum beyond the ligament of Treitz was noted (Figure 2). 

Exploratory laparotomy through a midline incision was planned. However, given the poor prognosis due to malignant carcinoma, the patient’s family decided not to go ahead with any further invasive treatment, including the jejunostomy tube insertion, total parenteral nutrition, thoracentesis, and endotracheal intubation. She was managed conservatively with nasogastric tube insertion and intravenous (IV) fluids and by keeping her nothing per oral (NPO). Her gastrointestinal symptoms improved, but she passed away within a week of the diagnosis due to worsening hypoxic respiratory failure.

## Discussion

SMA syndrome has been described by a variety of other terms in the literature, including Cast syndrome, arteriomesenteric duodenal obstruction, Wilkie syndrome, and chronic duodenal ileus [2]. The loss of mesenteric fat between the aorta and the SMA leads to the compression of the third portion of the duodenum. The third portion of the duodenum passes between the aorta and the SMA. The duodenum typically crosses anterior to the aorta at the level of the third lumbar vertebral body suspended by its attachment to the ligament of Treitz. The SMA arises from the anterior aspect of the aorta at the level of the L1 vertebral body. It is enveloped in fatty and lymphatic tissue and extends in a caudal direction at an acute angle into the mesentery.

Several factors can decrease the acuity of the angle between the aorta and the SMA. Most commonly, SMA syndrome has been associated with a debilitating illness such as malignancy, acquired immunodeficiency syndrome (AIDS) [3], burns [4], bariatric surgery [5], and anorexia nervosa [6]. Weight loss is not responsible for all cases. In younger patients, SMA syndrome is most commonly described following corrective spinal surgery for scoliosis [7]. Anatomic abnormalities (congenital or acquired) can also contribute.

The symptoms may be mild (e.g., early satiety or postprandial abdominal pain) in patients with a mild obstruction while those with severe obstruction may have severe nausea, bilious vomiting, and weight loss. Symptoms may be relieved when the patient is lying prone, in the left lateral decubitus, or in a knee-chest position. These positions remove tension from the mesentery and the SMA, opening the space between the SMA and the aorta.

Diagnostic evaluation should begin with abdominal radiographs. Although plain films are frequently nonspecific, they may reveal findings suggestive of proximal small bowel obstruction, for example, a distended stomach or dilated proximal duodenum. Upper gastrointestinal series usually demonstrate marked delay in the passage of the contrast from the duodenum into the more distal small bowel. The passage of contrast typically halts abruptly at the third portion of the duodenum [8]. CT and magnetic resonance (MR) arteriography are noninvasive diagnostic tools and provide additional anatomic detail such as the amount of intra-abdominal and retroperitoneal fat [9].

Generally, the following criteria can be used to diagnose SMA syndrome on imaging [10]: (a) duodenal obstruction with an abrupt cutoff in the third portion and active peristalsis, (b) an aortomesenteric artery angle of ≤25°, particularly if the aortomesenteric distance is ≤8 mm, and (c) low origin of SMA, high fixation of the duodenum by the ligament of Treitz, presence of SMA anomalies.

The management of SMA syndrome can be conservative or surgical. The goals of conservative treatment of SMA syndrome are the alleviation of obstructive symptoms and reversal of any precipitating factors. However, if surgery has altered the anatomy, the likelihood that conservative therapy will be successful is low. A nasogastric or orogastric tube should be inserted to decompress the dilated stomach and proximal duodenum. Nutritional support is usually required, at least in the initial stages, until patients are able (and willing) to increase oral intake. Enteral nutrition is preferred and often administered through a nasojejunal feeding tube placed distal to the obstruction. Total parenteral nutrition may be necessary if enteral feeding is not an option [11]. Once significant weight gain is noted, the diet may be advanced slowly. In adults who have a brief history of symptoms and in children who tend to present acutely, conservative management with nutritional support has good success [4]. The adult patient with more chronic symptoms is less likely to benefit from nutritional support alone. For these patients, correction of electrolytes and decompression with a short course of nutrition should be followed soon by surgical management if symptoms are not relieved [12].

## Conclusions

SMA syndrome is a rare cause of duodenal obstruction and is a diagnostically challenging condition. A delay in treatment is associated with life-threatening outcomes. A high index of suspicion should be maintained, especially in the settings of sudden weight loss. Initial resuscitation includes urgent nasogastric decompression and fluid electrolytes management. The surgical approach is employed if the patient fails to respond to conservative treatment. Nutritional support is an essential component of management and is usually required, at least in the initial stages, until the patient is able to increase oral intake.
